# BDNF enhances spontaneous and activity-dependent neurotransmitter release at excitatory terminals but not at inhibitory terminals in hippocampal neurons

**DOI:** 10.3389/fnsyn.2014.00027

**Published:** 2014-11-10

**Authors:** Yo Shinoda, Saheeb Ahmed, Binu Ramachandran, Vinita Bharat, David Brockelt, Bekir Altas, Camin Dean

**Affiliations:** ^1^Trans-synaptic Signaling Group, European Neuroscience InstituteGoettingen, Germany; ^2^Department of Applied Biological Science, Faculty of Science and Technology, Tokyo University of ScienceChiba, Japan

**Keywords:** brain-derived neurotrophic factor, neurotransmitter release, exocytosis, hippocampal neurons, excitatory/inhibitory

## Abstract

Brain-derived neurotrophic factor (BDNF) is widely reported to enhance synaptic vesicle (SV) exocytosis and neurotransmitter release. But it is still unclear whether BDNF enhances SV recycling at excitatory terminals only, or at both excitatory and inhibitory terminals. In the present study, in a direct comparison using cultured rat hippocampal neurons, we demonstrate that BDNF enhances both spontaneous and activity-dependent neurotransmitter release from excitatory terminals, but not from inhibitory terminals. BDNF treatment for 5 min or 48 h increased both spontaneous and activity-induced anti-synaptotagmin1 (SYT1) antibody uptake at excitatory terminals marked with vGluT1. Conversely, BDNF treatment did not enhance spontaneous or activity-induced uptake of anti-SYT1 antibodies in inhibitory terminals marked with vGAT. Time-lapse imaging of FM1-43 dye destaining in excitatory and inhibitory terminals visualized by *post-hoc* immunostaining of vGluT1 and vGAT also showed the same result: The rate of spontaneous and activity-induced destaining was increased by BDNF at excitatory synapses, but not at inhibitory synapses. These data demonstrate that BDNF enhances SV exocytosis in excitatory but not inhibitory terminals. Moreover, BDNF enhanced evoked SV exocytosis, even if vesicles were loaded under spontaneous vesicle recycling conditions. Thus, BDNF enhances both spontaneous and activity-dependent neurotransmitter release on both short and long time-scales, by the same mechanism.

## Introduction

Brain-derived neurotrophic factor (BDNF) is predominantly expressed in the CNS (Lewin and Barde, 1996) primarily by excitatory neurons, including pyramidal neurons and granule cells in the hippocampus (Ernfors et al., 1990; Dieni et al., 2012). The most well-characterized effect of BDNF is its enhancement of synaptic vesicle (SV) exocytosis (Lohof et al., 1993; Lessmann et al., 1994; Carmignoto et al., 1997; Sherwood and Lo, 1999; Tyler and Pozzo-Miller, 2001; Walz et al., 2006; Inagaki et al., 2008; Amaral and Pozzo-Miller, 2012) by TrkB receptor activation (Levine et al., 1995; Li et al., 1998; Tyler et al., 2006; Amaral and Pozzo-Miller, 2012). Specifically, this enhancement occurs via an increase in SV docking and release probability, which results in an increase in quantal neurotransmitter release and a greater number of recycling vesicles per terminal (Tyler and Pozzo-Miller, 2001; Tyler et al., 2006). This enhancement in SV exocytosis is thought to be associated with increased learning and memory (Yamada and Nabeshima, 2003; Cunha et al., 2010; Musumeci and Minichiello, 2011). Most efforts to investigate the BDNF-induced enhancement of SV exocytosis have been performed at excitatory terminals (Lohof et al., 1993; Lessmann et al., 1994; Carmignoto et al., 1997; Sherwood and Lo, 1999; Tyler and Pozzo-Miller, 2001; Walz et al., 2006; Amaral and Pozzo-Miller, 2012), however, the enhancement of neurotransmitter release at inhibitory terminals by BDNF has also been reported separately (Mizuno et al., 1994; Huang et al., 1999; Baldelli et al., 2002; Yamada et al., 2002; Ohba et al., 2005). BDNF can be released from both pre and post-synaptic sites (Hartmann et al., 2001; Matsuda et al., 2009) and can retrogradely enhance SV exocytosis (Dean et al., 2009). Thus, BDNF release from excitatory post-synaptic neurons could retrogradely affect inhibitory terminals.

Several mechanisms of enhanced SV exocytosis by BDNF have been proposed. In excitatory terminals, phosphorylation of synapsin I (Jovanovic et al., 2000), extra and intracellular Ca^2+^ (Amaral and Pozzo-Miller, 2012), activation of TRPC channels (Amaral and Pozzo-Miller, 2007, 2012), and Rab3a (Thakker-Varia et al., 2001), have all been reported to mediate BDNF-induced enhancement of excitatory SV fusion. On the other hand, changes in expression levels of GAD65 and vGAT have been proposed to mediate BDNF-induced enhancement of inhibitory SV fusion (Mizuno et al., 1994; Yamada et al., 2002; Ohba et al., 2005; Peng et al., 2010). Although BDNF-induced enhancement of SV fusion has been reported, how quickly this effect occurs following BDNF application, and the kinetics, degree of enhancement and mechanisms by which it occurs in excitatory vs. inhibitory terminals has not been directly compared. Moreover, it is not well understood if BDNF enhances both evoked and spontaneous SV using the same machinery.

In the present study, we carried out simultaneous analysis of evoked and spontaneous SV recycling in excitatory and inhibitory terminals of cultured rat hippocampal neurons and tested if SV recycling was enhanced by BDNF treatment, using two different optical methods. The first is static end-point visualization of SV recycling, using uptake of anti-SYT1 antibody in live neurons to visualize evoked and spontaneous recycling SVs by SYT1 immunostaining. The second is dynamic time-lapse imaging of SV exocytosis by FM1-43 destaining. We discovered that: (1) both short and long-term BDNF treatment enhanced SV fusion at excitatory but not inhibitory terminals; (2) both evoked and spontaneous SV fusion was enhanced in excitatory terminals, presumably by the same mechanism; and (3) BDNF treatment induces changes in the level of proteins associated with SV fusion, at excitatory but not inhibitory synaptic sites.

## Materials and methods

All research involving animals was approved by and done in accordance with the Institutional Animal Care and Ethics Committees of Goettingen University (T10.31), and in accordance with German animal welfare laws. All efforts were made to minimize the number of animals used and their suffering.

### Primary hippocampal cell culture and BDNF treatments

Dissociated hippocampal neurons were prepared according to previously described methods (Banker and Goslin, 1988) with minor modifications. Embryonic hippocampal neurons were obtained from timed pregnant rats at 18–19 days gestation. Cells were mechanically dissociated by trypsination and trituration. Neurons were plated onto 0.04% polyethyleneimine (PEI, Sigma) or 0.5 mg/ml poly-D-lysine (PDL, Sigma)-coated 12 mm glass coverslips (with grids for retrospective immunocytochemistry for FM1-43 experiments) in 24-well dishes at a density of 5–10 × 10^4^ cells/well. Cultures were maintained in GlutaMAX and B27-supplemented Neurobasal medium containing 100 U/ml penicillin/streptomycin (all from Invitrogen,) at 37°C in a 5% CO_2_ humidified atmosphere. BDNF (R&D Systems) at a final concentration of 100 ng/ml was added to culture media at 13 DIV for 48 h or at 15 DIV for 5 min.

### SYT1 antibody uptake

Neurons were incubated with a guinea pig polyclonal anti-SYT1 antibody (Synaptic Systems) at 1:800 in 47 mM KCl (evoked) or 4 mM KCl (basal) Tyrode solution for 5 min, and then in basal Tyrode solution containing the same antibody concentration for 5 additional min. After washing with basal Tyrode solution for 10 min, a mouse monoclonal anti-SYT1 antibody (Synaptic Systems) was loaded in the presence of 1 µM Tetrodotoxin (TTX) in basal Tyrode solution for 10 min for spontaneous uptake. After washing with basal Tyrode solution for 10 min, cells were fixed by 4% paraformaldehyde/0.1 M phosphate buffer. All incubations except fixation were performed at 37°C. The basal Tyrode solution contained (in mM): 150 NaCl, 4 KCl, 2 CaCl_2_, 2 MgCl_2_, 10 Glucose and 10 4-(2-hydroxyethyl)-1-piperazineethanesulfonic acid (HEPES). The evoked condition high potassium Tyrode solution contained (in mM): 107 NaCl, 47 KCl, 2 CaCl_2_, 2 MgCl_2_, 10 Glucose and 10 HEPES. The pH of the solutions was adjusted to 7.3 and osmolality to 310 mOsmol.

### FM dye imaging

Fluorescence microscopy was performed using a Carl Zeiss Axio Observer Z1 inverted epifluorescence microscope equipped with 100× 1.3 NA objective and filter set for FM1-43 (472/30 nm bandpass excitation, 511 nm longpass beamsplitter and 582/75 nm bandpass emission). Images were acquired with a Photometrics Evolve CCD camera controlled by MetaMorph software (Molecular Devices). Coverslips of cultured neurons were transferred to an imaging chamber that contains electrodes for electrical stimulation (Warner Instruments). For FM1-43 unloading experiments using high extracellular potassium concentrations for depolarization, neurons were loaded with FM1-43 by incubation with 10 µM FM1-43 (Invitrogen) in high potassium HEPES-buffered saline (HK-HBS) containing (in mM) 107 NaCl, 47 KCl, 2 CaCl_2_, 2 MgCl_2_, 10 HEPES and 10 D-Glucose, adjusted to pH 7.3. Before unloading experiments, coverslips were placed in low potassium HBS (LK-HBS) containing (in mM) 150 NaCl, 4 KCl, 2 CaCl_2_, 2 MgCl_2_, 10 HEPES and 10 D-Glucose, adjusted to pH 7.3. Unloading of FM1-43 by high potassium solution was achieved by perfusion of HK-HBS. For FM1-43 loading by field electrical stimulation, neurons were incubated in 10 µM FM1-43 in LK-HBS and stimulated at 20 Hz for 30 s. For FM1-43 loading by spontaneous activity, neurons were incubated in 10 µM FM1-43 in LK-HBS containing 1 µM TTX for 10 min. After washing the loaded neurons for 5 min in LK-HBS, unloading of FM1-43 was achieved by field stimulation at 20 Hz for 80 s. All solutions were supplemented with 10 µM 6-cyano-7-nitroquinoxaline-2,3-dione (CNQX) and 50 µM DL-2-amino-5-phosphonovaleric acid (APV) to prevent recurrent excitation. All experimental procedures were performed at room temperature. Images were acquired at a rate of 1 Hz. After destaining, neurons were immediately fixed for retrospective imunocytochemistry. To standardize imaging conditions, identical solutions (blocking, antibody, FM dye, depolarization, and wash) from a single mix in the same tube were used for control, 5 min, and 48 h BDNF treated excitatory and inhibitory samples. For microscopy identical magnification, neutral density filters, lamp intensity, excitation and emission wavelengths, camera gain, bin size, and exposure time were used for all samples. Fluorescence intensities in the 5 min and 48 h treated samples were normalized to control in each experiment, and control and treated samples were imaged alternately with identical acquisition settings in each experiment.

ROIs with synaptic characteristics; round/oval in shape, an optical size of approximately 1 µm in diameter, and distributed along neuronal processes, were selected manually. The bleach/spontaneous release rate was less than 0.25% per frame for all conditions. Background fluorescence remaining after complete destaining (the point at which the destain curve slope becomes zero) was subtracted to normalize complete destain to zero. FM dye destaining curves were fit by single exponential decays after subtracting background using Excel software. Goodness-of-fit, tested by R-squared, was greater than or equal to 90%.

### Immunocytochemistry and image analysis

For immunocytochemistry cultured neurons were fixed with 4% paraformaldehyde/0.1 M phosphate buffer and then washed with PBS. After blocking and permeabilizing in antibody buffer (2% donkey serum, 0.1% Triton X-100, and 0.05% sodium azide in 2× PBS), cells were incubated with primary antibody in antibody buffer at 4°C overnight or room temp for 2 h. Cells were then washed 3× 3 min in PBS, incubated with secondary antibody in antibody buffer at room temp for 2 h and washed again 3× 3 min in PBS. Coverslips were then mounted with Fluoromount Plus (Diagnostic Biosystems) and examined using 100× objective on a Zeiss Axiovert 200 epifluorescence microscope equipped with a cooled CCD camera (RETIGA-SRV, Q-Imaging, Canada). To standardize imaging conditions, identical blocking and antibody solutions from a single mix in the same tube were used for control, 5 min, and 48 h BDNF treated excitatory and inhibitory samples. For microscopy identical magnification, neutral density filters, lamp intensity, excitation and emission wavelengths, camera gain, bin size, and exposure time were used. Fluorescence intensities in 5 min and 48 h treated samples were normalized to control in each experiment. Intensity of fluorescence of syt-1 uptake, TrkB, or phospho-TrkB at excitatory vs. inhibitory synapses was analyzed using MetaMorph software (Molecular Devices) by thresholding the vGluT or vGAT signal to mark excitatory vs. inhibitory synapses, respectively. The number of excitatory and inhibitory synapses was counted along 40 µm secondary dendrites or 40 µm axon, respectively. Antibodies used were: mouse and guinea pig syt1, rabbit and guinea pig vGluT1, rabbit and mouse vGAT, VAMP2, P/Q alpha-1A VGCC, syt3, syt9 (Synaptic Systems), SMI-312 (Covance), synapsin1, Rab3a (kindly provided by Reinhard Jahn, Max Planck Institute for Biophysical Chemistry, Goettingen), syt12, syt17 (Abcam), MAP2 and TrkB (Millipore), and phospho-TrkB (kindly provided by Moses Chao, New York University, USA). Secondary antibodies used were Alexa 488, 546 and 647 (Invitrogen) conjugated.

### Hippocampal slice culture and western blotting

Hippocampal slice cultures were prepared as described previously (Fuller and Dailey, 2007) from P5 rat pups. The hippocampus was removed from the brain and 400 µm thick slices were cut transversely from both hippocampi in ice-cold Hank’s balanced salt solution (HBSS) containing glucose, using a tissue chopper (Stoelting). BDNF (100 ng/ml) was added to the slice culture media at DIV14 for 5 min or 48 h incubation. After treatment with 100 ng/ml BDNF for 5 min or 48 h, slices were washed once and homogenized in 320 mM sucrose, 4 mM HEPES-KOH, pH 7.4, in a glass-Teflon homogenizer with 10 strokes at 900 rpm. Protein concentration was determined using a BCA kit (Calbiochem, cat. no. 71285-3) and equal protein amounts were subjected to SDS-PAGE followed by immunoblotting. Antibodies used for Western blotting were mouse monoclonal anti-Rab3a and anti-tubulin, and mouse monoclonal anti-VAMP2 (all from Synaptic Systems).

## Results

### BDNF enhances SYT1 antibody uptake at excitatory, but not inhibitory terminals

To determine if BDNF enhances SV fusion at both excitatory and inhibitory terminals in neurons, we used optical measurements to directly compare SV recycling in both excitatory and inhibitory terminals in the same experimental paradigm in response to BDNF treatment.

**Figure 1:**
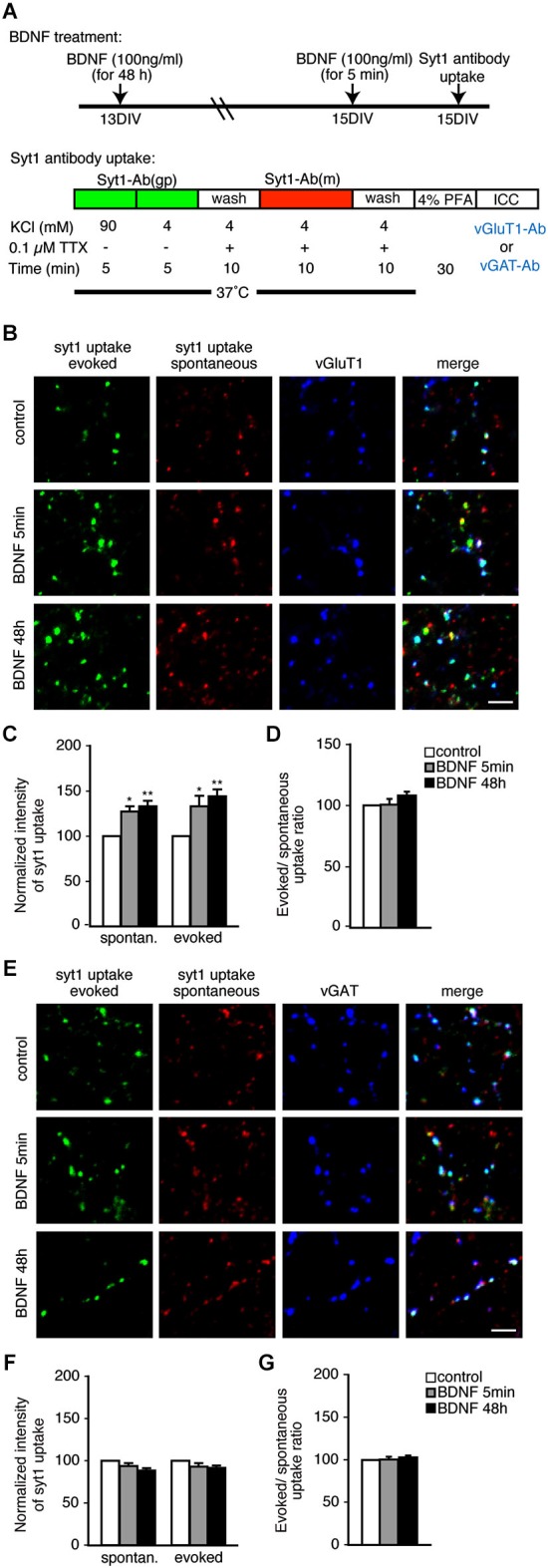
**Both evoked and spontaneous uptake of anti-SYT1 antibody is enhanced by BDNF treatment in excitatory but not inhibitory terminals. (A)** Schematic of the anti-SYT1 antibody uptake experiment. **(B)** Representative immunofluorescence images of evoked and spontaneous anti-SYT1 antibody uptake in excitatory terminals. **(C)** Statistical analysis of normalized fluorescence intensity of both evoked and spontaneous anti-SYT1 antibody uptake in control (white), 5 min (gray) and 48 h (black) BDNF treatments in vGluT1 positive terminals. **(D)** Ratio of evoked and spontaneous anti-SYT1 antibody uptake. **(E)** Representative immunofluorescence images of evoked and spontaneous anti-SYT1 antibody uptake in inhibitory terminals. **(F)** Statistical analysis of both evoked and spontaneous anti-SYT1 antibody uptake in inhibitory terminals in control (white), 5 min (gray) and 48 h (black) BDNF treatments. **(G)** Ratio of evoked and spontaneous anti-SYT1 antibody uptake. Scale bars = 5 µm.

We first tested the effects of BDNF using an anti-SYT1 antibody uptake assay and subsequent immunostaining with the excitatory and inhibitory marker proteins vGluT1 and vGAT to visualize SV recycling. Two different BDNF treatment times—5 min and 48 h—were tested in both spontaneous and evoked conditions (Figure 1A). Day *in vitro* (DIV) 13 hippocampal neurons were treated with BDNF for 48 h. For short-term 5 min BDNF treatment, 15 DIV neurons were used. At 15 DIV, a guinea pig anti-SYT1 antibody against the lumenal domain of SYT1 was applied in the presence of 47 mM KCl to depolarize neurons and induce anti-SYT1 antibody uptake to visualize evoked SV recycling. After washout of the SYT1 antibody, a mouse anti-SYT1 lumenal domain antibody was applied in the presence of 0.1 µM TTX to suppress action potentials and allow spontaneous anti-SYT1 antibody uptake, to visualize spontaneous SV recycling (Figure 1A). After washout of the second SYT1 antibody, cells were fixed and subsequently immunostained with anti-vGluT1 or anti-vGAT antibodies to observe the amount of evoked and spontaneous SV recycling (assayed by anti-SYT1 fluorescence intensity) in excitatory and inhibitory terminals. This method allows a direct comparison of evoked and spontaneous uptake of anti-SYT1 antibodies by recycling vesicles in excitatory and inhibitory terminals (Figures 1B–G). Quantitation of anti-SYT1 fluorescence intensity revealed a significant enhancement of both evoked and spontaneous anti-SYT1 antibody uptake in excitatory terminals in both 5 min and 48 h BDNF treatment groups (Figure 1C; *n* = 41, 26 and 33 terminals for 5 coverslips each of control, 5 min and 48 h BDNF-treated samples, respectively, * *p* < 0.05, ** *p* < 0.01, one-way ANOVA *post hoc* Tukey-Kramer test). The degree of enhancement of evoked and spontaneous uptake was identical in both 5 min and 48 h BDNF treatments (Figure 1D). In contrast, neither evoked nor spontaneous anti-SYT1 antibody uptake was observed in inhibitory terminals in the same BDNF treatments (Figures 1E–G; *n* = 40, 24 and 33 terminals for 5 coverslips each of control, 5 min and 48 h BDNF-treated samples, respectively).

### BDNF does not change the number of SVs per terminal or synapses per unit length

**Figure 2:**
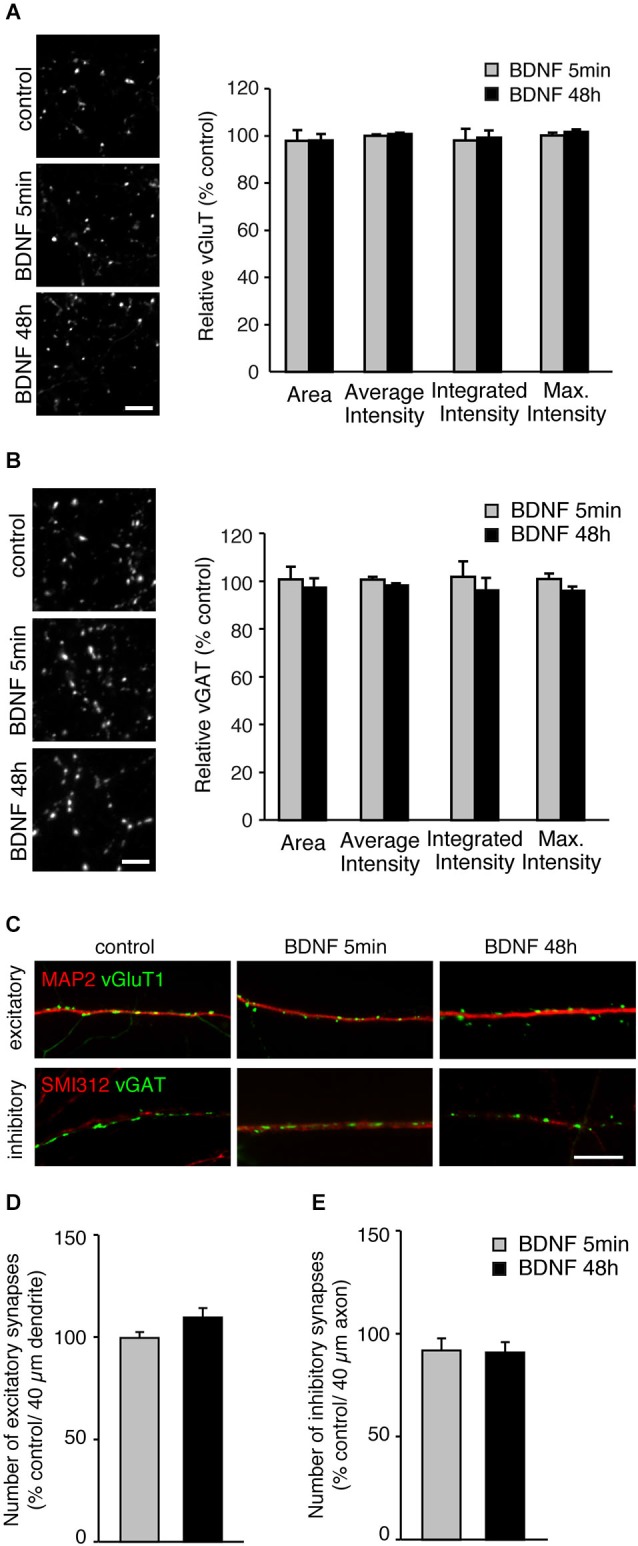
**BDNF treatment does not alter total SV number or number of excitatory or inhibitory synapses. (A)** Representative immunofluorescence images of vGluT1 immunostaining (left) and quantified data of vGluT1 fluorescence intensity in control and each BDNF treated group normalized to control (right). Scale bar = 5 µm. **(B)** Representative immunofluorescence images of vGAT immunostaining (left) and quantified data of vGAT average, integrated and maximum fluorescence intensity in control and each BDNF treated group normalized to control (right). Scale bar = 5 µm. **(C)** Representative immunofluorescence images of excitatory (vGluT1) and inhibitory (vGAT) synapses along dendrites (MAP2) and axons (Smi312), respectively. Scale bar = 10 µm. **(D)** Quantification of number of excitatory synapses along dendrites. **(E)** Quantification of number of inhibitory synapses along axons. Statistical significance in all panels was analyzed by one-way ANOVA.

A possible mechanism by which BDNF could enhance SV release is by increasing the number of SVs per terminals. To test this, we analyzed the fluorescence intensity of vGluT1 and vGAT immunopositive puncta in individual terminals in BDNF treated cultures compared to control (Figures 2A,B). The fluorescence intensity of vGluT1 and vGAT immunopositive puncta is thought to correlate with the number of SVs in excitatory and inhibitory synaptic terminals, respectively. Compared with control neurons, cultures treated for 5 min or 48 h with BDNF showed identical fluorescence intensity of both vGluT1 (Figure 2A; *n* = 60, 78 and 66 terminals for 12 coverslips each of control, 5 min and 48 h BDNF-treated samples, respectively) and vGAT (Figure 2B; *n* = 78, 64 and 75 terminals for 12 coverslips each of control, 5 min and 48 h BDNF-treated samples, respectively) immunopositive puncta. This suggests that BDNF does not change the total number of SVs per terminal, although we cannot exclude that SV number is reduced while protein content per vesicle is increased, or vice versa, resulting in no apparent change in fluorescence intensity of the proteins tested.

BDNF has been reported to affect not only SV recycling but also synapse number (Vicario-Abejón et al., 1998; Rico et al., 2002; Bamji et al., 2006). No direct association between BDNF-induced enhancement of SV fusion in excitatory terminals and alterations in excitatory synapse number has been reported. However, a change in cellular excitability elicited by BDNF-induced enhancement of SV fusion could indirectly affect the number of excitatory and inhibitory synapses. To test this, we analyzed the number of vGluT1 and vGAT immunopositive puncta along dendrites (marked with MAP2) and axons (marked with SMI-312). The number of excitatory vGluT1 positive terminals along secondary dendrites identified by MAP2 staining, were identical in control and BDNF treated neurons (Figures 2C,D; *n* = 18, 19 and 20 for 4 coverslips each of control, 5 min and 48 h BDNF-treated samples, respectively, significance determined by one-way ANOVA). Because the number of inhibitory synapses along dendrites varies, for example between proximal and distal dendrites, we quantified the number of vGAT positive puncta along axons marked with SMI-312. The number of inhibitory vGAT positive terminals along axons was also unaltered by BDNF treatment (Figures 2C,E; *n* = 16, 16 and 18 for 4 coverslips each of control, 5 min and 48 h BDNF-treated samples, respectively, significance determined by one-way ANOVA), similar to the results obtained for excitatory terminal number. Thus, the BDNF treatments we performed did not affect excitatory or inhibitory synapse number. However, we cannot exclude that BDNF application promotes dendritic/ axonal outgrowth, branching or neuronal survival, which may result in an overall increase in synapse number.

### BDNF enhances FM1-43 destaining from excitatory, but not inhibitory terminals

**Figure 3:**
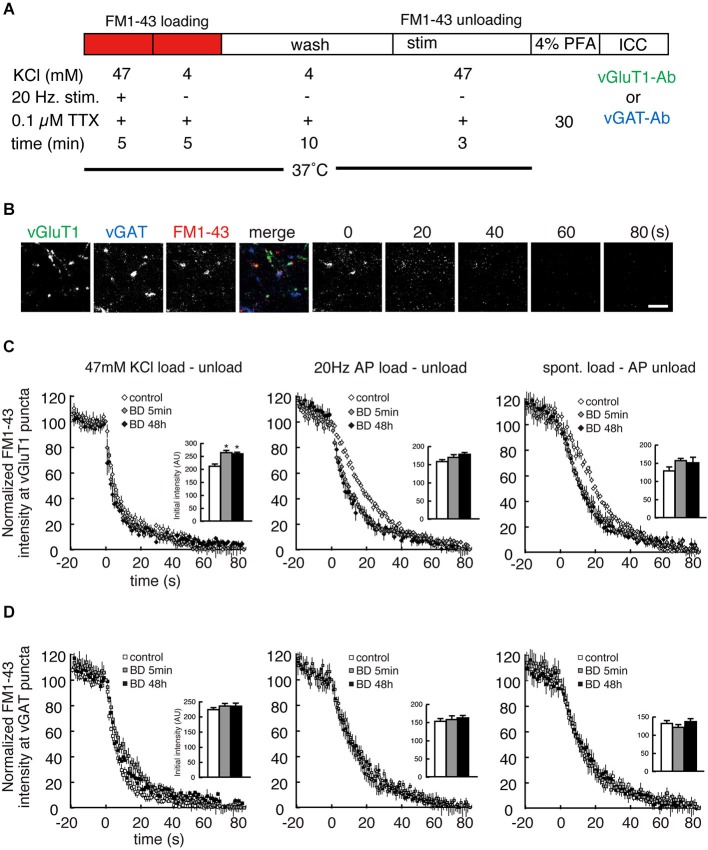
**Both evoked and spontaneous destaining of FM1-43 dye is enhanced by BDNF treatment in excitatory but not inhibitory terminals. (A)** Schematic of the FM1-43 staining and destaining experiment. **(B)** Representative time-lapse image frames of FM1-43 destaining and *post hoc* vGluT1 and vGAT immunostaining. Scale bar is 5 µm. **(C)** Time course of FM1-43 fluorescence destaining using the indicated loading and unloading protocols in excitatory terminals. Data were normalized to the initial fluorescence intensity of each FM1-43 fluorescent punctum. Insets indicate the initial value of FM1-43 fluorescence in loaded presynaptic terminals. **(D)** Time course of FM1-43 fluorescence destaining using the indicated loading and unloading protocols in inhibitory terminals. Data were normalized to the initial fluorescence intensity of each FM1-43 fluorescent punctum. Inset graphs indicate the initial value of FM1-43 fluorescence in loaded presynaptic terminals.

The anti-SYT1 antibody uptake experiments showed that BDNF treatment significantly enhanced SV recycling in excitatory terminals but not inhibitory terminals. However, this relatively “static” end-point experiment does not show how BDNF might alter the kinetics of SV fusion. Moreover, antibody uptake results only report exocytosis indirectly, based on the assumption that the rates of exocytosis and endocytosis are coupled. We therefore next tested SV exocytosis in real time by measuring the release kinetics of FM1-43 from SVs by live time-lapse imaging followed by *post hoc* immunostaining to identify excitatory and inhibitory terminals. After BDNF treatment, FM1-43 was loaded by three different protocols: 47 mM KCl, 20 Hz electrical stimulation and spontaneous loading in the presence of 0.1 µM TTX (Figure 3A). The former two loading protocols reflect evoked loading and the third reflects spontaneous loading. After washout, subsequent unloading of FM1-43 was carried out by the same protocol as that used for loading, except in the test of spontaneous loading followed by 20 Hz electrical stimulation unloading. All neurons imaged by time-lapse were fixed and *post hoc* immunostaining was performed with anti-vGluT1 and anti-vGAT to distinguish excitatory and inhibitory terminals (Figure 3B). FM1-43 unloading experiments showed a significant increase in FM1-43 destaining kinetics in excitatory terminals in cultures treated with BDNF for 5 min or 48 h, consistent with the anti-SYT1 antibody uptake results (Figure 3C: *n* = 8–15; 20 Hz *τ* = 26.1 ± 1.6 (control), 14.2 ± 1.2 (5 min), 14.1 ± 1.8 (48 h), *p* < 0.001 (***) for control vs. 5 min or 48 h BDNF; spont. *τ* = 28.2 ± 1.0 (control), 19.5 ± 2.7 (5 min), 17.4 ± 2.3 (48 h), *p* < 0.01 (**) for control vs. 5 min or 48 h BDNF, one-way ANOVA *post hoc* Tukey-Kramer test; R-squared ≥ 90%). No enhancement of FM1-43 dye destaining kinetics was observed in the 47 mM KCl load-unload experiment, most likely because this relatively strong stimulation results in a maximal rate of destain (47 mM KCl *τ* = 10.3 ± 1.4 (control), 10.3 ± 2.6 (5 min), 8.7 ± 0.9 (48 h)). However, the initial loaded FM1-43 fluorescence intensities were significantly increased in BDNF treated cultures compared to control (Figure 3C, upper right inset graph). In contrast, no enhancement of FM1-43 load or destaining kinetics was observed in inhibitory terminals following BDNF treatment in spontaneous, 47 mM KCl or 20 Hz electrical stimulation load-unload conditions (Figure 3D: *n* = 8–15; 47 mM KCl *τ* = 8.2 ± 1.0 (control), 12.1 ± 2.0 (5 min), 9.5 ± 0.7 (48 h); 20 Hz *τ* = 20.4 ± 2.5 (control), 16.7 ± 2.8 (5 min), 19.1 ± 3.6 (48 h); spont. *τ* = 17.6 ± 2.2 (control), 17.0 ± 2.7 (5 min), 20.1 ± 3.2 (48 h); R-squared ≥ 90%).

### TrkB distribution and phosphorylation following BDNF addition is identical at excitatory and inhibitory synapses

**Figure 4:**
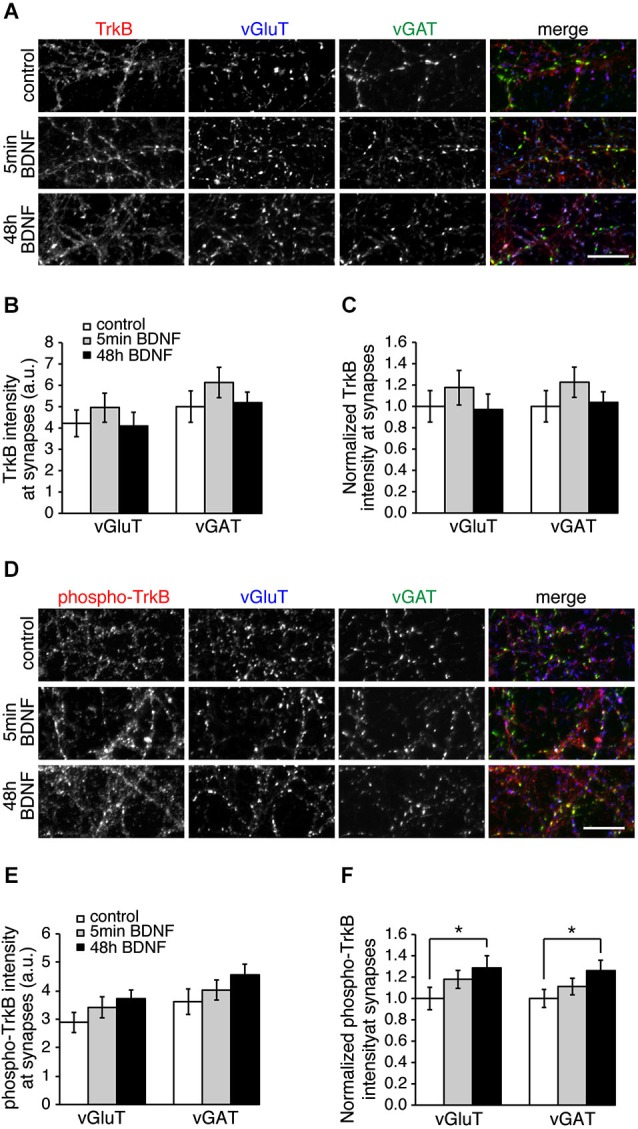
**Levels of TrkB and phosphorylated TrkB are the same at excitatory and inhibitory synapses. (A)** Hippocampal neurons in control conditions and treated with BDNF for 5 min or 48 h and immunostained for TrkB, vGluT and vGAT. **(B)** Quantitation of arbitrary fluorescent units and normalized **(C)** TrkB intensity at excitatory (vGluT-positive) or inhibitory (vGAT-positive) synapses in control and BDNF-treated conditions. **(D)** Control and BDNF-treated hippocampal neurons immunostained for phospho-TrkB, vGluT and vGAT. **(E)** Quantitation of arbitrary fluorescent units and normalized **(F)** phospho-TrkB intensity at excitatory (vGluT-positive) or inhibitory (vGAT-positive) synapses. Scale bars = 10 µm.

The specific effect of BDNF on excitatory SV recycling could be caused by more TrkB receptors at excitatory synapses, or more TrkB phosphorylation at excitatory synapses. To test this we immunostained control and BDNF-treated hippocampal cultures with TrkB or phospho-TrkB antibodies and vGluT and vGAT to mark excitatory and inhibitory synapses, respectively (Figure 4). TrkB receptors were enriched at synaptic sites, but were present at both excitatory and inhibitory synapses in equal amounts (Figures 4A–C). TrkB phosphorylation increased significantly following BDNF treatment for 48 h, but did so in equal amounts at both excitatory and inhibitory synapses (Figures 4D–F; *n* = 9 images from three different cultures for each condition, * *p* < 0.05, Student’s *t*-test). Thus the distribution or phosphorylation of TrkB receptors themselves is not the cause of differences in BDNF-induced SV recycling at excitatory vs. inhibitory synapses.

### BDNF treatment decreases VAMP2 and Rab3a levels in excitatory terminals

**Figure 5:**
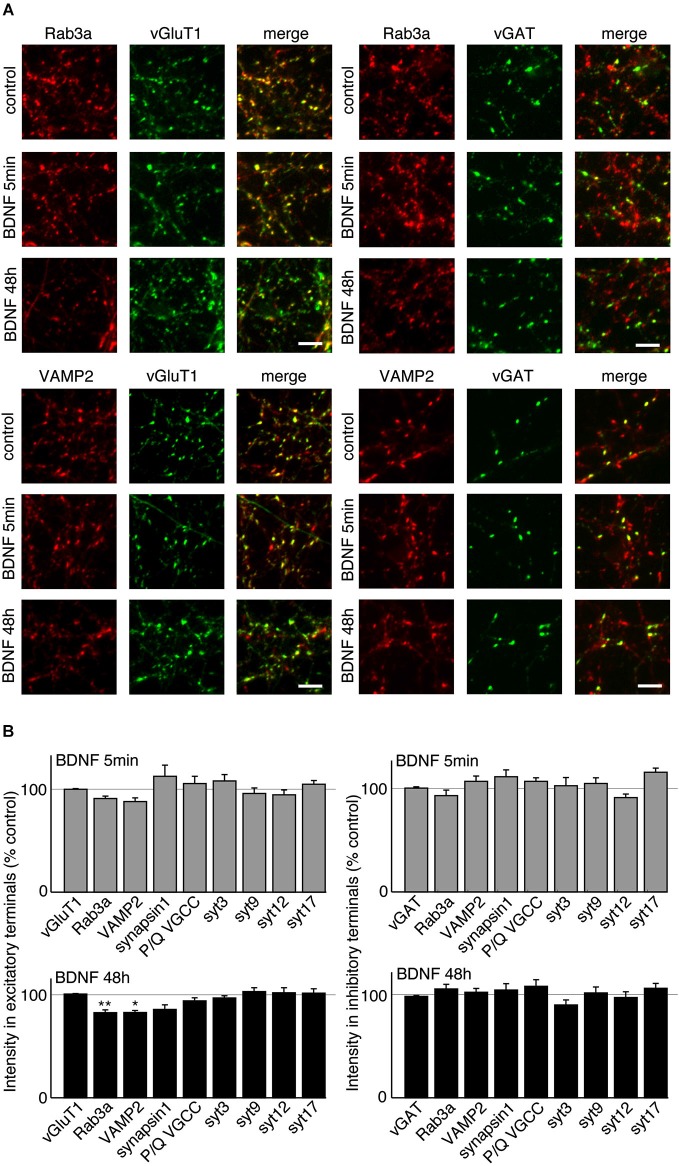
**VAMP2 and Rab3a are decreased by long-term BDNF treatment in excitatory but not inhibitory terminals. (A)** Representative immunofluorescence images of Rab3a and VAMP2 in excitatory and inhibitory terminals in control conditions and following treatment with BDNF for 5 min or 48 h. **(B)** Quantified immunofluorescence of synaptic proteins in excitatory or inhibitory terminals in 5 min and 48 h BDNF treated groups compared to control. Scale bars = 5 µm.

**Figure 6:**
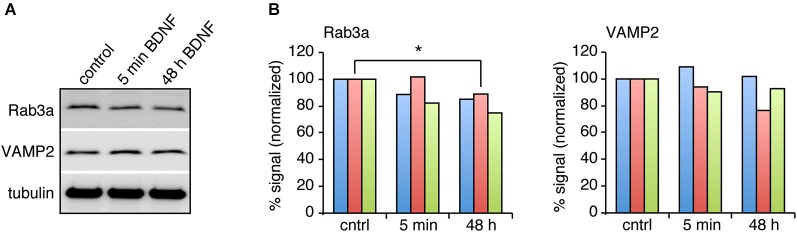
**Rab3a protein levels are decreased in response to 48 h BDNF treatment. (A)** Western blot of Rab3a and VAMP2 in hippocampal slices treated with BDNF for 5 min or 48 h compared to control. **(B)** Quantitation of Rab3a and VAMP2 levels in Western blots of homogenate from hippocampal slices from three different samples, normalized to tubulin levels.

BDNF treatment could affect SV fusion by modifying the association of proteins with SVs or active zones on short time scales, or by changing protein expression levels over longer time periods (Tartaglia et al., 2001; Alder et al., 2003). We therefore examined the levels of proteins reported to be associated with BDNF-induced SV release in excitatory and inhibitory terminals by quantitative immunostaining. We hypothesized that the levels of some of these proteins may be altered specifically in excitatory terminals in response to BDNF treatment. BDNF treated cultures were fixed and immunostained with antibodies against SV related proteins together with anti-vGluT1 and vGAT to identify excitatory and inhibitory terminals. The fluorescence intensity of each antibody was quantified in excitatory and inhibitory terminals. For most antibodies tested, there was no change in levels at excitatory or inhibitory synapses following BDNF treatment. However, the fluorescence intensity of Rab3a and VAMP2 in excitatory terminals was slightly (but not significantly) decreased following 5 min BDNF treatment, and significantly decreased following 48 h BDNF treatment (Figures 5A,B; *n* = 12 each for all proteins in control, 5 min and 48 h BDNF-treated samples, * *p* < 0.05, ** *p* < 0.01, one-way ANOVA *post hoc* Tukey-Kramer test). In addition, we tested if the total protein levels of Rab3a or VAMP2 were decreased in hippocampal slices in response to BDNF treatment. We found a significant decrease in Rab3a following 48-h treatment with BDNF (Figure 6; * *p* < 0.05, Student’s *t*-test).

## Discussion

Our main finding in the present study is that BDNF enhances SV recycling at excitatory synapses but not at inhibitory synapses. In addition, our findings lead to three conclusions: (1) both short and long-term BDNF treatment enhance SV fusion and recycling at excitatory but not inhibitory terminals; (2) BDNF enhances both evoked and spontaneous SV release, suggesting a similar mechanism in each case; and (3) long-term BDNF treatment changes the levels of proteins associated with BDNF-induced SV release at excitatory but not inhibitory terminals.

In excitatory terminals, distinct SV pools that undergo evoked vs. spontaneous fusion have been reported (Sara et al., 2005; Fredj and Burrone, 2009), although this is debated (Groemer and Klingauf, 2007). We found that BDNF has similar effects on both spontaneous and evoked excitatory SV recycling. In addition, BDNF enhanced evoked release even when vesicles were loaded with FM dye under spontaneous recycling conditions, suggesting that it operates by a similar mechanism to enhance these two types of fusion events. Vesicle pools are more heterogeneous at glutamatergic than GABAergic synapses (Moulder et al., 2007), suggesting that excitatory SVs are more malleable and capable of being modified by BDNF.

What is the mechanism by which BDNF enhances neurotransmitter release at excitatory synapses and not at inhibitory synapses? TrkB is present at both synapse types (Figure 4; Swanwick et al., 2004) making it unlikely that TrkB itself plays a role. We found significantly altered levels of the SV associated proteins Rab3a and VAMP2 at excitatory synapses and not at inhibitory synapses in response to BDNF treatment, suggesting that these proteins play a role in modifying SV recycling in response to BDNF. Both Rab3a and VAMP2 were significantly decreased at excitatory synapses by BDNF treatment. Rab3a dissociates from SV during exocytosis (Fischer von Mollard et al., 1991) and thus might be expected to be reduced at presynaptic terminals that are highly active. Our results are consistent with early reports that more exocytosis occurs in Rab3a knockouts compared to wild-type synapses (Geppert et al., 1997). A BDNF-induced decrease in Rab3a specifically at excitatory synapses would then be expected to increase exocytosis at these synapses, and this is indeed what we observe. VAMP2 also disperses in the plasma membrane following fusion (Degtyar et al., 2013), which may result in an apparent decrease in fluorescence at more active synapses. But both Rab3a and VAMP2 proteins are present at both excitatory and inhibitory synapses, and are thus unlikely to initiate the BDNF-induced effect. A proteomics comparison of excitatory and inhibitory SV associated proteins yielded relatively few proteins that were specifically localized to one vesicle sub-type (Grønborg et al., 2010), suggesting that vesicle proteins themselves may not cause the BDNF-induced effect. It may, nonetheless, be interesting to immunoprecipitate vGluT1 and vGAT-containing vesicles from cultures treated with BDNF to test if SV composition changes in one vesicle type and not the other.

A more likely but as yet unexplored possibility for the mechanism by which BDNF affects excitatory SV recycling is via a post-synaptic protein present at excitatory synapses and not inhibitory synapses. Neuroligin1, for example, is specifically localized to excitatory post-synaptic sites, and can retrogradely enhance the size of the recycling SV pool at apposed presynaptic terminals (Wittenmayer et al., 2009). BDNF may bind to post-synaptic TrkB receptors and signal via neuroligin1 to presynaptic sites to enhance SV recycling. Alternatively, it is possible that different times and concentrations of BDNF could affect excitatory vs. inhibitory SV recycling differently, and the time and concentration we chose predominantly affects excitatory synapses. This seems unlikely, however, since we specifically chose times and concentrations of BDNF that are commonly reported to enhance both excitatory and inhibitory synapses.

Our results are similar to those reporting BDNF-induced enhancement of excitatory SV recycling (Lohof et al., 1993; Lessmann et al., 1994; Carmignoto et al., 1997; Sherwood and Lo, 1999; Tyler and Pozzo-Miller, 2001; Walz et al., 2006; Amaral and Pozzo-Miller, 2012), but differ from reports of enhanced inhibitory SV recycling (Mizuno et al., 1994; Huang et al., 1999; Baldelli et al., 2002; Yamada et al., 2002; Ohba et al., 2005). However, many reports of inhibitory effects of BDNF implicate SV recycling indirectly. For example, an observed increase in GABA content in the striatum after BDNF injections (Mizuno et al., 1994) could be caused by increased GABAergic release, or by increased synthesis. Similarly, BDNF-induced increases in miniature or evoked GABAergic release (Baldelli et al., 2002; Yamada et al., 2002) could be caused by an increase in GABAergic release probability or an increase in GABAergic synapse number. This raises the possibility that the inhibitory effects of BDNF are caused by alternative mechanisms, some of which may be post-synaptic (Brünig et al., 2001; Wardle and Poo, 2003). Tanaka et al. (1997), for example, reported suppression of inhibition by BDNF entirely by a postsynaptic mechanism. It is also likely, given the bulk of the literature, that BDNF affects excitatory SV recycling at mature synapses, but affects GABAergic transmission specifically during development and synapse maturation (Huang et al., 1999; Baldelli et al., 2002; Rico et al., 2002; Yamada et al., 2002). In any case, there is no question that BDNF affects both excitatory and inhibitory synapse function.

It is important to note that dissociated hippocampal cultures likely do not represent native *in vivo* hippocampal circuits. The effects of BDNF could depend on the activity and connectivity state of the neurons in a circuit. For example, BDNF enhances evoked excitatory SV recycling when both pre and post-synaptic cells are excitatory, but not at excitatory-inhibitory synapses (Schinder et al., 2000). In addition, in organotypic hippocampal cultures, BDNF increases synapse numbers (Tyler and Pozzo-Miller, 2001; Amaral and Pozzo-Miller, 2007), but in studies in dissociated culture, including ours, BDNF does not change synapse numbers (Sherwood and Lo, 1999; Bolton et al., 2000). The reduced dissociated culture system we used, however, is an advantage because it allows us to assess intrinsic cellular effects of BDNF at individual synapses using imaging approaches. In addition, although exogenous BDNF is widely used to study its function, it does not reflect the native source of BDNF. Endogenous BDNF is likely released from specific sites in specific amounts at specific times to affect synapse function. In this study we used exogenous BDNF to isolate the effects of BDNF irrespective of its site of release.

The diverse and intricate roles of BDNF reported in a variety of cellular and synaptic processes, using different experimental techniques, makes it difficult to discern specific effects. Our goal in the present study was to directly test one of the most commonly reported effects of BDNF, i.e., the enhancement of SV recycling, where it is unclear if this occurs at excitatory synapses, inhibitory synapses, or both. We used two different assays to directly examine effects of both short and long-term BDNF treatment on SV recycling, bypassing potential post-synaptic effects and changes in synapse number that cannot be excluded in electrophysiological experiments. Our combination of anti-SYT1 antibody uptake and FM dye destaining experiments, which reflect the size of the SV recycling pool and release probability at synapses (Branco et al., 2008; Tokuoka and Goda, 2008) showed that BDNF specifically affects SV recycling at excitatory synapses and not at inhibitory synapses. This direct comparison more clearly defines the specific role of BDNF in a distinct pathway—SV recycling—that affects synapse strength and underlies BDNF-induced changes in circuit function and plasticity.

## Conflict of interest statement

The authors declare that the research was conducted in the absence of any commercial or financial relationships that could be construed as a potential conflict of interest.
